# Study of the Role of the Tumor Microenvironment in Ovarian Cancer (MICO): A Prospective Monocentric Trial

**DOI:** 10.1002/cnr2.70242

**Published:** 2025-06-04

**Authors:** Martina Arcieri, Eleonora Capezzali, Stefano Restaino, Sara Pregnolato, Laura Mariuzzi, Alessandro Mangogna, Maria Orsaria, Angelica Tulisso, Silvia Tonon, Maria De Martino, Miriam Isola, Lorenza Driul, Carlo Pucillo, Giovanni Scambia, Barbara Frossi, Giuseppe Vizzielli

**Affiliations:** ^1^ Clinic of Obstetrics and Gynecology “Santa Maria Della Misericordia” University Hospital, Azienda Sanitaria Universitaria Friuli Centrale Udine Italy; ^2^ Department of Medicine (DMED) University of Udine Udine Italy; ^3^ Department of Obstetrics and Gynecology University of Sassari Sassari Italy; ^4^ Institute of Pathology Academic Hospital Udine Italy; ^5^ Gynecologic Oncology Unit Fondazione “Policlinico Universitario A. Gemelli IRCCS,” Catholic University of the Sacred Heart Rome Italy; ^6^ Institute of Obstetrics and Gynecology Catholic University of the Sacred Heart Rome Italy

**Keywords:** organoids, ovarian cancer, tumor microenvironment

## Abstract

**Background:**

Ovarian cancer (OC) is one of the most aggressive tumors requiring new therapeutic approaches. Immunotherapy represents an opportunity, but to date, OC patients do not appear to benefit from current protocols. A better understanding of the composition of the tumor microenvironment (TME), especially in its immune components, could unveil mechanisms of immune suppression in a useful way to predict response to therapies and develop new therapeutic approaches.

**Method:**

The MICO (tumor MICroenvironment of Ovarian cancer) study is a single‐center observational study. Starting from peritoneal biopsy of high‐grade serous ovarian carcinoma (HGSOC), the purpose of the MICO study is to generate tumor patient‐derived organoid (PDOs) cultures and evaluate the concordance between in vitro platinum‐based chemotherapy sensitivity and in vivo sensitivity. Simultaneously, we will characterize through multiparameter cytofluorimetric analysis the composition of the OC TME, focusing on B lymphocytes and mast cells whose roles in ovarian cancer remain controversial and underinvestigated. Furthermore, patients experiencing recurrence will be longitudinally followed to monitor changes in the TME composition and the responsiveness of PDOs to in vitro stimulation with drugs.

**Discussion:**

The association between the composition of the TME, the reactivity of the PDOs, and patients' disease progression will be analyzed to identify whether specific subpopulations of tumor‐infiltrating immune cells could be predictive factors of the disease outcomes. The comparison of molecular profiles, in vitro response to drugs, and clinical‐pathological data will allow the definition of a pattern capable of predicting the response of the primary tumor for the identification of those patients who may benefit from specific treatment.

**Strengths and Limitations:**

The results of our study could help to better understand the OC behavior, may have implications for the development of effective immunotherapy and targeted pharmacological therapies for epithelial OC in a personalized medicine perspective. This will be a monocentric trial with an involvement of only 43 patients, so further studies will need to confirm our results.

**Trial Registration:**

The clinical trial has been registered at Clinical‐Trials.gov with the identifier NCT06272240 on 02/14/2024

**Abbreviations**
BMI: body mass index
CRS: chemotherapy response score
HGSOC: high grade serous ovarian carcinoma
IDS: interval debulking surgery
MCs: mast cells
NACT: neoadjuvant chemotherapy
OC: ovarian cancer
PARP‐I: PARP‐inhibitors
PBMCs: peripheral blood mononucleated cells
PDOs: patient‐derived organoid
PDS: primary debulking surgery
PDX: patient‐derived xenografts
PI: predictive index
TIL: tumor‐infiltrating lymphocytes
TME: tumor microenvironment

## 1 Introduction

Despite the significant advances made by modern oncology, ovarian cancer (OC) remains one of the most aggressive tumors requiring new therapeutic approaches. The mechanisms underlying the development of OC are complex and heterogeneous and exhibit diverse features depending on the patient. A detailed understanding of these molecular mechanisms is essential for the development of innovative therapies tailored to the specific patient.

Over the past decades, advances in molecular and cellular biology have provided remarkable insights into the intricate dynamics underlying OC progression, revealing the pivotal role of the tumor microenvironment (TME) in shaping disease outcomes. TME is composed of a multi‐faceted assembly of stromal cells, immune cells, fibroblasts, blood vessels, and extracellular matrix components that together create a nurturing niche for tumor cells to thrive [1, 2, 3]. TME is also an attractive niche for microbial growth, and recently, it has been assumed that intra‐tumoral microbiota play a crucial role in shaping the TME in several tumors, including OC [4]. Among the components of the TME, immune infiltrate has garnered increasing attention due to its multifunctional roles in influencing tumor growth, metastasis, and therapeutic responses. It includes T, B, and natural killer cells, but also macrophages, dendritic cells, and mast cells that can take part in the orchestration of both the immune response against the tumor and the tumor's evasion mechanisms [5]. The immunosuppressive factors secreted by tumor cells, coupled with the recruitment of regulatory immune cells, contribute to the establishment of a microenvironment that hampers effective anti‐tumor immune responses. Conversely, mounting evidence suggests that a robust and well‐coordinated immune response can restrain tumor growth and metastasis development, highlighting the potential of immune‐based therapies in improving clinical outcomes for different cancer types, including OC [6, 7, 8, 9, 10, 11, 12, 13].

Lymphocytes are important components of the TME and include B and T cells. Indeed, multiple studies have shown that the presence of CD3^+^ and CD8^+^ tumor‐infiltrating lymphocytes (TIL) is associated with prolonged survival [6, 7, 8, 9, 10, 11, 12, 13]. Zhang et al. demonstrated that the presence of intratumoral T cells correlates with better clinical outcomes in advanced OC [14]. Depending on the degree of infiltration of T lymphocytes (CD3^+^ and CD8^+^) and using a standardized scoring system, tumors can be classified as “hot” or “cold.” “Hot” cancers exhibit high T cell infiltration, whereas “cold” tumors have few or none, or they are limited to the periphery of the tumor [15]. CD8^+^ TILs do not operate in isolation: tumors containing CD8^+^ TILs are often additionally infiltrated by CD20^+^ B cells, but their role in OC remains elusive [16]. Among the cells of the innate branch of the immune system present in the TME of OC, mast cells represent a controversial element, with authors supporting both anti‐tumoral and pro‐tumoral roles [17, 18]. A recent study showed that an abundance of naïve B cells and activated mast cells in the immune TME correlated with a worse prognosis [19]. Therefore, extensive knowledge of mast cells in OC is mandatory.

OC is a very heterogeneous tumor: the various shades of the TME composition, both between different OCs and within different sites of the same tumor, confirm it. In general, boosting the immune response against cancer cells represents a valuable therapeutic tool, but to date, OC patients do not appear to benefit from current immunotherapy protocols. We believe that deciphering the composition of the immune infiltrate in OC and pointing out the interplay among tumor and resident immune cells is necessary to better understand the behavior of OC.

Previous scientific studies have contributed to explaining the pathogenesis of OC using cell lines and animal models; however, the new frontier in biomedical research is represented by organoids, three‐dimensional cell culture systems obtained from a tissue fragment that reproduces the fundamental properties of the original tissue in vitro [20, 21, 22]. Cultures of epithelial organoids, which are found to be genetically and phenotypically stable when derived from both healthy and diseased tissues, can provide a valuable model for in vitro study of various pathologies [20]. They have paved the way for new large‐scale experimental techniques for the identification of genetic mutations that may provide specific sensitivities to anticancer drugs, thus enabling the definition of increasingly personalized treatments. Although organoid technology currently has some limitations, which can be attributed to some factors, including a lack of standardized protocols, heterogeneity amongst organoid preparations, and an absence of complexity and full physiological representation, (PDO) [23] have already found numerous applications in regenerative medicine and can be used to advance knowledge in the field of OC [24].

Starting from the patient's biopsy, the purpose of the MICO study (Study of the Role of the Tumor Microenvironment in Ovarian Cancer) is to characterize in detail the composition of the OC TME, focusing on B lymphocytes and mast cells that have been poorly investigated so far. Simultaneously, PDOs will be generated for each patient and will be employed for in vitro assays to evaluate the tumor's resistance to chemotherapy and to PARP inhibitors (PARP‐I). Furthermore, patients experiencing recurrence will be longitudinally followed to monitor changes in the TME composition and the responsiveness of PDOs to in vitro treatment with drugs. The association between the composition of the TME, the reactivity of the PDOs, and the survival rate of the patients will be analyzed in order to identify whether specific subpopulations of tumor‐infiltrating immune cells are predictive factors of the disease outcome. Finally, PDOs will be stored and used in the future for in vitro studies to find possible interactions among tumor cells and immune cells.

The comparison of molecular profiles, in vitro response to drugs, and clinical‐pathological data will allow the definition of patterns capable of predicting the response of the primary tumor for the identification of those patients who may benefit from treatment.

## 2 Methods

### 2.1 Trial Design

The MICO trial is a prospective monocentric study conducted at the University of Udine, Italy. Ethical approval was obtained from the Ethics Committee (CEUR FVG n. 17 380).

**FIGURE 1:**
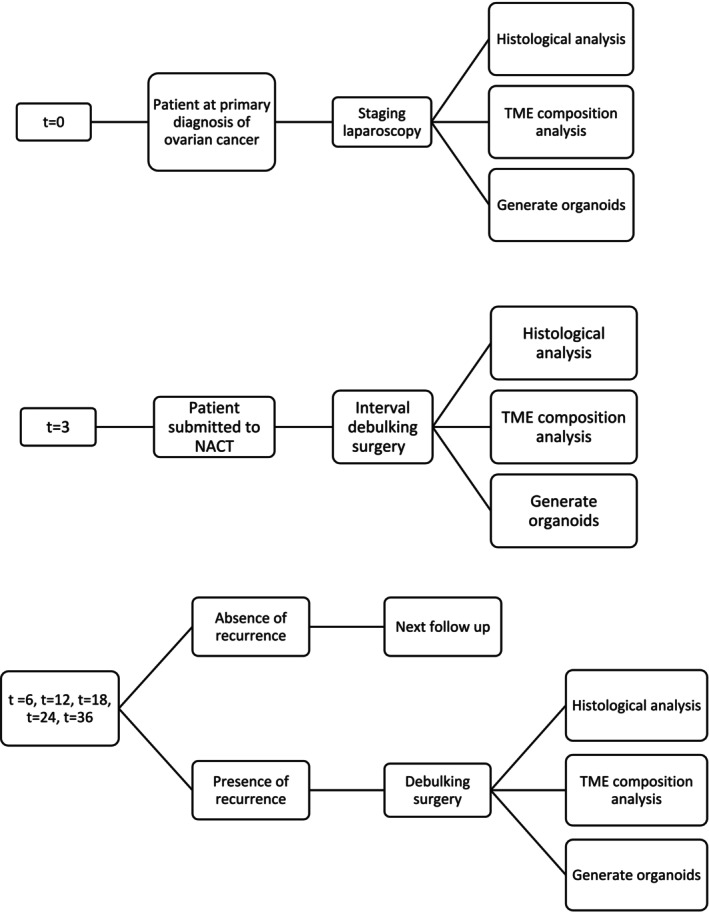
Flowchart of MICO trial. t is expressed in months. NACT = neoadjuvant chemotherapy; TME = tumor microenvironment.

The MICO trial was registered on ClinicalTrials.gov under NCT06272240 identifier on 02/14/2024. Patients with International Federation of Gynecology and Obstetrics (FIGO) stages III–IV HGSOC (epithelial high grade serous ovarian carcinoma) will be enrolled. Peritoneal biopsies will be collected at the time of surgery (*t* = 0), and fresh tissue samples will be sent to the Pathology Department. Tumor tissue will be obtained at the time of diagnosis before any other treatment (Figure 1).

Clinical data collection of HGSOC patients will include age, menopausal status, Body Mass Index (BMI), BRCA mutational status, history of breast cancer, preoperative CA125, type of surgery, predictive index (PI) score, 2014 FIGO stage [25] and residual tumor.

All HGSOC patients will be triaged for staging laparoscopy (LPS) to obtain a histological diagnosis and to provide tumor load assessment through the PI score. The intraoperative evaluation will consist of histological diagnosis of HGSOC at frozen section and verification of diffusion intras‐abdominal disease.

Patients with LPS‐PI < 10 will undergo open primary debulking surgery (PDS); if PI was ≥ 10, patients were referred to neoadjuvant chemotherapy [26, 27].

After 3–4 cycles of neoadjuvant chemotherapy (NACT), in the presence of reduced tumor load, patients will undergo interval debulking surgery (IDS). During IDS, peritoneal biopsies will be collected at the time of surgery (*t* = 3) and fresh tissue samples will be sent to the Department of Medicine, University of Udine.

The pathologist will evaluate the chemotherapy response score (CRS), a three‐level pathological response scoring system on omental specimens, which can predict prognostic outcomes in patients with advanced epithelial OC undergoing neoadjuvant chemotherapy [28, 29].

At *t* = 0 and *t* = 3, the removed tissue will be sent to the Pathology Department, where it will be selected by the pathologist, who will collect a fragment of tumor tissue and will divide it into three parts: one for the identification of cells composing the TME (through multiparameter cytofluorimetric analysis), one for histological diagnosis, and one for organoid generation.

**FIGURE 2:**
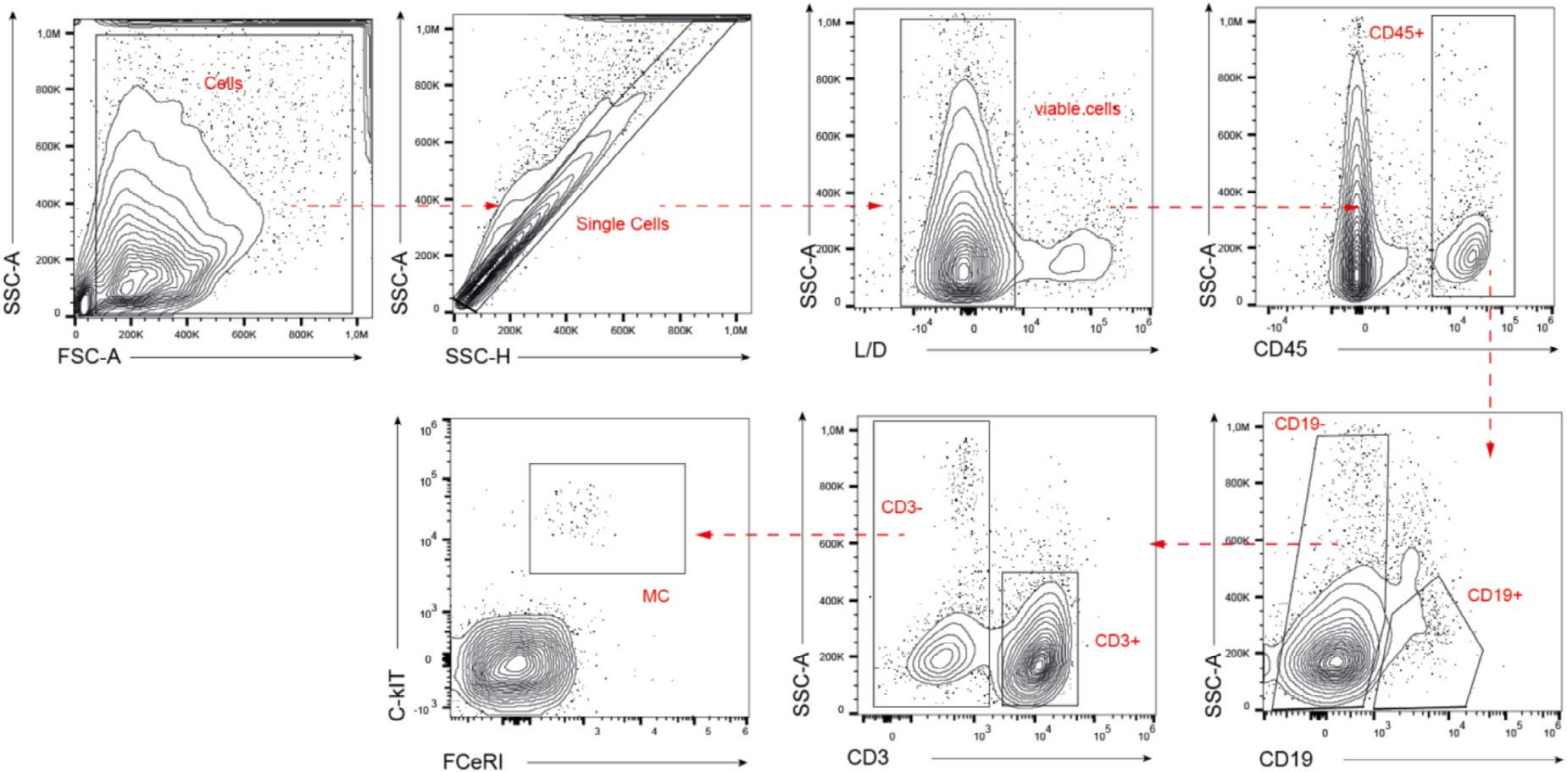
Proposed gating strategies for the identification of B cells, T cells, and mast cells (MC) among the CD45+ cells infiltrating the TME. Cells will be selected in the FSC‐A versus SSC‐A plot, then only singlets (SSC‐H vs. SSC‐A), viable (negative for L/D in L/D vs. SSC‐A plot) and CD45^+^ cells (CD45 vs. SSC‐A) will be taken into consideration for the analysis. B cells (CD19^+^) will be estimated directly among CD45^+^ cells, while T cells (CD3^+^) will be selected in the CD19^−^ gate. Finally, MC (c‐kit^+^ FceR^+^) will be visualized from the CD3^−^ gate. B cells, T cells, and MC percentages will be calculated on CD45^+^ cells. The gating strategy will be manually drawn by an expert flow cytometrist and applied to all samples.

**FIGURE 3:**
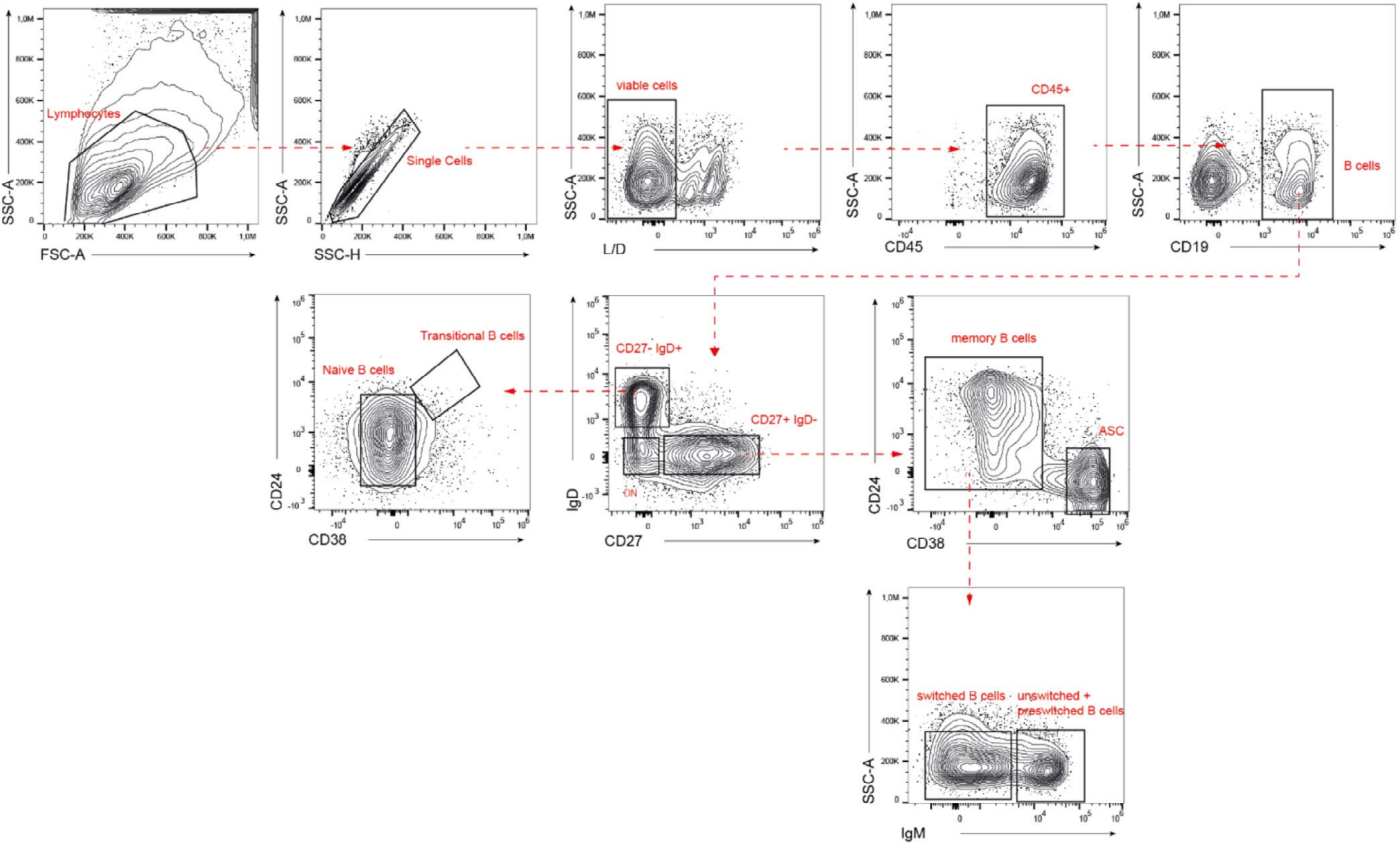
Gating strategy for the identification of the B cell subpopulations. Lymphocytes will be selected in the FSC‐A versus SSC‐A plot, then only singlets (SSC‐H vs. SSC‐A) and viable cells (negative for L/D in L/D vs. SSC‐A plot) will be taken into consideration for the analysis. For tissue samples, CD45^+^ (CD45 vs. SSC‐A) cells will be selected, while in blood samples this marker will not be used, directly selecting B cells for their positivity for CD19 (CD19 vs. SSC‐A). Among B cells, different subtypes will be analyzed, as extensively described in the text. The gating strategy will be manually drawn by an expert flow cytometrist and applied to all samples.

Through multiparameter cytofluorimetric analysis we will determine the main immune cell populations that infiltrate the tumor and, in parallel, the detailed immunophenotype of tumor infiltrating B cells and in the peripheral blood will be determined. Three different panels of antibodies will be used: one to identify the main populations of immune cells (CD45^+^) including B lymphocytes (CD19^+^), T cells (CD3^+^), and mast cells (FceRI^+^/cKIT^+^) in the TME (Figure 2). The other two panels will be employed for the complete characterization of B cells in the TME and in peripheral blood (Figure 3). Both Ab combinations allow the identification of transitional B cells (CD19^+^ CD24^++^ CD38^++^ CD27^−^ IgD^+^ IgM^+^), naïve B cells (CD19^+^ CD24^+/low^ CD38^+/low^ CD27^−^ IgD^+^ IgM^+^), memory unswitched + pre‐switched B cells (CD19^+^ CD24^+^ CD38^−^ CD27^+^ IgD^−/lo^ IgM^+^), memory switched B cells (CD19^+^ CD24^+^ CD38^−^ CD27^+^ IgD^−^ IgM^−^), plasmablasts (CD19^+^ CD24^−^ CD38^++^ CD27^+^ IgD^−/^ IgM^−^), and Double Negative (DN) B cells (CD19^+^ CD27^−^ IgD^−^). Figures 2 and 3 show the gating strategy employed to identify the main populations of immune cells in the TME (Figure 2) and to identify the B subpopulations in the TME (Figure 3) applied to the same representative HGSOC patient, respectively. Moreover, the concomitant evaluation of the positivity to CD21 on B cell subpopulations present in the peritoneal biopsy will allow us to characterize the activation status of the B cell subpopulations in the TME [30]. While the activation status of circulating B cells will be evaluated through the staining of CD71, a valuable marker of peripheral B cells proliferation [31].

**FIGURE 4:**
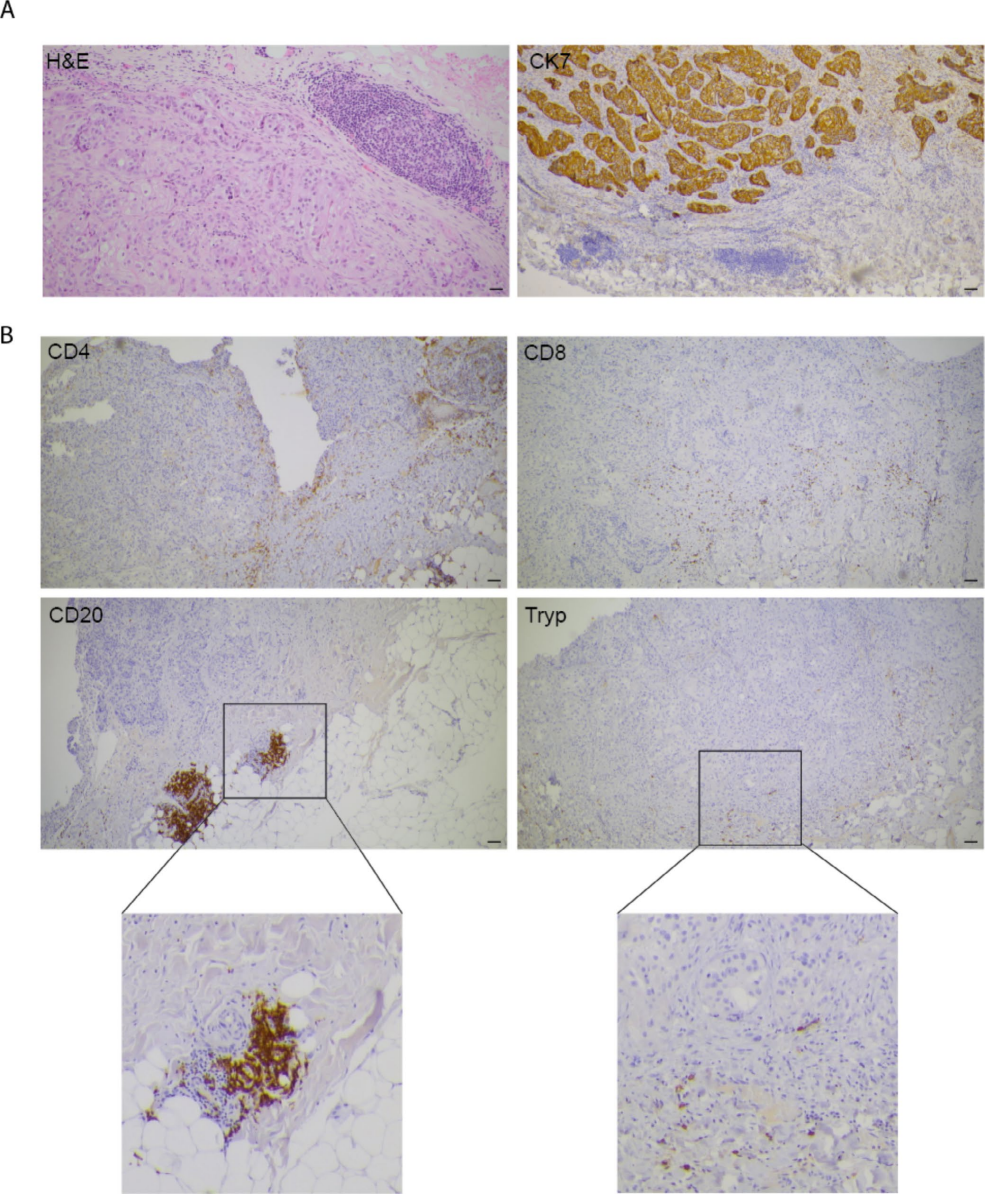
Example of immunohistological identification of immune markers in HGSOC. (A) Hematoxylin and eosin (H&E) staining and Cytokeratin‐7 (CK7) immunohistochemistry of peritoneal biopsy of one representative HGSOC patient. (B) Representative immunohistological images used to identify CD4+ T cells, CD8+ T cells, CD20+ B cells, and tryptase+ mast cells in the TME of the same representative HGSOC patient. All images taken at 5× magnification; 200 μm scale bar.

The data on immune cell numbers and phenotypes obtained from cytofluorimetric analysis of the digested biopsies will be confirmed by histological evaluation of the presence of tumor‐infiltrating B, T, and mast cells in the samples from the same patient. As reported in Figure 4 histological images of immune cells infiltrating the TME of a representative HGSOC subject show detectable CD4^+^ and CD8^+^ T cells as well as CD20^+^ B cells that are mostly peritumoral and organized in lymphocyte aggregates. Tryptase+ mast cells are also evident and appear focally distributed at tumour periphery.

The histological analysis will serve to corroborate the data obtained from the cytofluorimetric analysis, while also providing information on the spatial distribution of immune cells in the tissue and potentially providing insight into the co‐localization of B and mast cells in the tumor. Our preliminary observations showed the presence of tertiary lymphoid structures in patients with a high CRS score after NACT (data not shown), suggesting the active role of the lymphoid response in patients. Furthermore, double staining for mast cell tryptase and specific cytokines (TNF‐α, IL‐17, IL‐10, …) will be performed to characterize the number and distribution of mast cells in the TME as well as their activation state. The number of mast cells and their cytokine profile will be correlated with tumor grade (according to the FIGO scoring system) and response to NACT. These observations will determine whether mast cells possess a plastic phenotype dependent on the inflammatory environment and will shed light on the functions of MC in OC progression.

The in vitro expanded PDOs will be studied to identify molecular changes common to all analyzed ovarian tumors and/or unique to each individual patient. Briefly, the mutational status of the PDO will be compared to that of the corresponding tumor tissue; then PDO cultures will be evaluated for the expression of markers of ovarian tissue (cytokeratin 7, PAX8, and WT‐1) by immunohistochemistry or confocal microscopy. Moreover, organoids' susceptibility to drugs (Carboplatin and Parp‐I Olaparib) will be estimated through the CellTiter‐Glo 3D Cell Viability Assay. Eventually, co‐cultures of PDO and B cells (isolated from peripheral blood) or mast cells (using LAD2 or HMC‐1 mast cell lines) will be set up to investigate how the presence of B cells or mast cells influences the PDO response to drugs.

**FIGURE 5:**
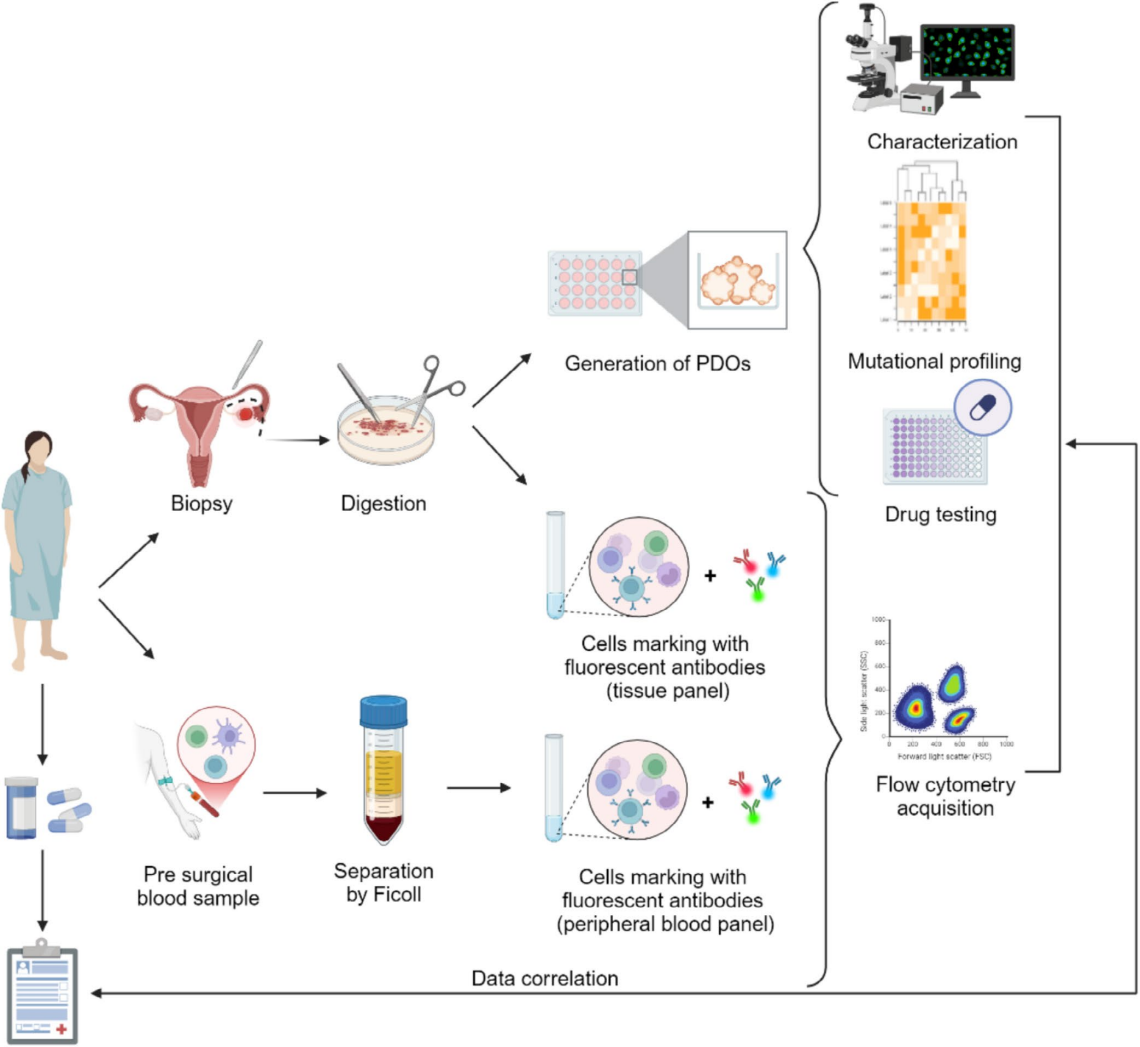
Experimental steps of MICO trial (created with Biorender.com).

Aliquots of PDOs, circulating or infiltrating immune cells and/or RNA isolated from them will be preserved for gene expression studies. Culture supernatants will be kept at −80°C for metabolite detection (Figure 5).

Six months after surgery (*t* = 6), and at subsequent 6‐month intervals (*t* = 12, *t* = 18, *t* = 24, and *t* = 36), patients will undergo regular follow‐up according to the clinical protocol. In case of recurrences requiring further surgical intervention, TME and blood will be reanalyzed, and new PDOs cultures will be generated. The phenotype of immune cells infiltrating the tumor and PDOs reactivity will be compared to those obtained at *t* = 0. This will make it possible to monitor changes over time in the composition of TME and the behavior of the PDOs.

Participation in the study will be requested during scheduled visits to the Obstetrics‐Gynecology Department of ASUFC or during outpatient visits. All data obtained during the study will be processed and treated exclusively in pseudo‐anonymized form for research purposes and will not be used for other purposes. Each collected sample will be coded with a progressive number.

### 2.2 Participants

Eligibility criteria are described in Table 1. The MICO study will enroll patients with advanced HGSO cancer.

**TABLE 1:** MICO study inclusion and exclusion criteria.

Inclusion criteria	Exclusion criteria
Patients ≥ 18 and < 80 years old	Patients < 18 or > 80 years old
Pathologic diagnosis of HGSOC	Ongoing or suspended immunosuppressive therapy within the last 6 months
FIGO Stages III–IV	Congenital or acquired immunodeficiency
Patient having signed the consent to participate in the study	Immunosuppressive state
	BMI > 30
	Chemotherapy for another malignancy in the last 12 months
	Diagnosis of HGSOC without surgery

The control group will include women who underwent risk reduction bilateral salpingo‐oophorectomy and peritoneal biopsies due to genetic predisposition to OC. Exclusion criteria will be the same as for HGSO patients, excluding the diagnosis of HGSOC. Women with a diagnosis of occult peritoneal, ovarian, or tubal cancer and serous tubal intraepithelial carcinoma on definitive pathologic examination will also be excluded.

### 2.3 Study Objectives and Endpoints

The primary endpoint of the MICO study will be to evaluate the concordance between in vitro platinum‐based chemotherapy sensitivity and in vivo sensitivity.

In more detail:

1. Organoids generation in vitro system to evaluate tumoral cells' response to carboplatin treatment.
2. Patient's clinical response monitoring. We will record platinum‐free interval (defined as interval expressed in months between the last dose of platinum‐based chemotherapy and the recurrence or progression of the disease), progression‐free survival (calculated from the first surgery until the first disease progression or the last follow‐up) and overall survival (defined as the length of time from the date of first surgery to death or the date last seen). The disease recurrence or progression will be evaluated according to RECIST1.1 [32]. Relapsed patients will be divided according to response to platinum treatment as resistant (platinum‐free interval 1–6 months of last platinum cycle) and partially platinum‐sensitive/platinum‐sensitive (platinum‐free interval > 12 months).

The model will be considered concordant if PDOs of platinum‐resistant patients will be viable on day 5 after carboplatin addition and if PDOs of platinum‐sensitive/partially platinum‐sensitive patients will not be viable (see Section 2.8).

The secondary endpoints will be:

1. Characterize the composition of the immune TME in order to understand if there are differences between tumor‐infiltrating immune subpopulations among patients, differences that could be predictive of early recurrence and/or different responses to therapy, focusing on immune populations still poorly studied (such as B cells for acquired immunity and mast cells for innate immunity).
2. Organoids generation in vitro system to evaluate tumoral cells' viability in response to PARP‐I treatment.
3. Association of TME composition, organoid response to drugs, and clinical evidence to identify predictors of response to therapy.
4. To compare the TME of HGSOC patients with the TME of the control group.
5. To develop a platform that allows in vitro analysis of the tumor followed by the identification of personalized therapy for each patient and, in case of recurrence, to monitor over time changes in the composition and responsiveness of the patient's TME.

As exploratory endpoints, immune cells from patients that have responded to platinum therapy and from patients that have relapsed within 18 months would be investigated at a single‐cell level throughout single‐cell RNA‐seq and CITE‐seq approaches, multi‐omics techniques for obtaining transcriptome and surface‐proteome information, respectively, at a single‐cell level. This will help in the identification of altered molecular pathways in B cells, mast cells, and other cells of interest in the TME, to possibly discover novel therapeutical targets.

### 2.4 Sample Size

The sample size was calculated in order to evaluate the concordance between in vitro platinum‐based chemotherapy sensitivity and in vivo sensitivity.

From literature, this concordance is 0.80 with traditional patient‐derived xenografts [33]; we expect a concordance of 0.95 using PDOs [34]. A sample size of 93 patients will allow us to detect a true value of Cohen's Kappa of 0.95, with a power of 80% and an allocation of the two groups of 1:1.

Ninety‐three control patients will also be enrolled to compare the number of CD45+ cells at baseline. This sample size will allow estimating an effect size of CD45+ cell number of less than 0.3 between the control and case groups, with an alpha of 5% and power of 80%.

[32, 33].

### 2.5 Blood Samples

Blood samples are collected before the surgery and peripheral blood mononucleated cells (PBMCs) are isolated using Ficoll Paque Plus (GE Healthcare Pharmacia, Cat No: 17‐1440‐03) and SepMate 50 mL Tubes (Stem Cell Technologies, Cat No: 154500) following the manufacturer's instructions. The supernatant is thoroughly discarded, the pellet is resuspended in 1 mL of Bambanker (NIPPON Genetics Europe GmbH, Cat No: BB02), and stored at −80°C.

### 2.6 Patient Tissue Sample Manipulation

Biopsies from patients with FIGO stages III–IV HGSOC will be collected from patients treated at the Obstetrics‐Gynecology Department of Ospedale Santa Maria della Misericordia, Udine, Italy. HGSOC tissue samples are gently washed with PBS in a 60 mm Petri dish (Corning, Cat. No: 430166) to remove excess blood, and then moved to a new Petri dish with AdDF^+++^ culture medium (Advanced DMEM/F12 (ThermoFisher, Cat. No: 12634010) containing L‐Glutamine (Merck, Cat. No: G7513‐100ML), 10 mM HEPES (Merck, Cat. No: 63264‐100ML‐F) and Penicillin–Streptomycin (Life Technologies, Cat. No: 15070063). Tissue is then minced with a scalpel into small fragments of 1–2 mm^2^ and digested in 5 mL AdDF+++ containing 5 μM RHO/ROCK pathway inhibitor (Y‐27632, Tocris) and 100 μg/mL Primocin (Invivogen, Cat. No: ant‐pm‐1), 1 mg/mL Collagenase type IV (ThermoFisher, Cat. No: 17104019) on an orbital shaker at 37°C for 1 h 30 min.

The cell suspension is then filtered through a 100uM Cell Strainer (Starlab, Cat. No: CC8111‐0072) placed on a 50 mL tube and washed with 5‐10 mL of AdDF^+++^ culture medium and centrifuged at 1200 rpm for 5 min at room temperature. The pellet is resuspended with AdDF^+++^ and cells, diluted in 1× Trypan Blue (Sigma‐Aldrich, Cat. No: T6154), are counted using a Neubauer chamber. Cells are used for cytofluorimetric analyses and organoid generation.

### 2.7 Patient‐Derived Organoid Generation and Characterization

For organoid generation, 30,000 cells/well of a pre‐warmed 24‐well (Greiner, Cat. No: 662160) are embedded in a mix of 1/3 of AdDF^+++^ culture medium and 2/3 Matrigel (Corning, Cat. No: 356231). The plate is placed at 37°C for 20 min to allow the Matrigel to polymerize before being overlaid with 500uL of complete growth factor medium and incubated at 37°C.

Complete growth factor medium consists of AdDF^+++^ culture medium supplemented with 100 ng/mL recombinant mEGF (ThermoFisher, Cat. No: 315‐09‐1 mg), 0.5uM A83‐01 (Tocris, Cat. No: 2939), 1 mM Nicotinamide (Sigma‐Aldrich, Cat. No: N0636), 1× N2 supplement (Life Technologies, Cat. No: 17502048), 1× B27 Supplement (Life Technologies, Cat. No: 17504044).

For cryopreservation of the cells, resuspend the pellet in the appropriate volume of a mix composed of 60% AdDF^+++^, 30% FBS, and 10% DMSO (AppliChem, Cat. No: APA36720250). The mutational profile of the PDOs, which may include mutations in BRCA1, BRCA2, TP53, and so on, will be evaluated using the Sanger method and subsequently compared with those of the parental tumor tissue.

### 2.8 Organoids Viability Assay

Organoids will be released from Matrigel by using Cell Recovery Solution (Corning) and collected by centrifugation. Organoid pellet was combined with Matrigel in a 1–2 ratio and seeded in a 96‐well plate at 20 μL per well. After Matrigel solidification 80 μL of complete medium is added. Carboplatin) will be added 2 two days after embedding, and cell viability will be measured after 5 days using Cell Titer‐Glo (Promega) following manufacturers' indications. Data will be analyzed with GraphPad Prism 9.0 software to calculate IC_50_.

For co‐culture experiments, B cells are isolated from peripheral blood using the B cell isolation kit (Milteny) and 100,000 cells will be added to each 96‐well containing organoids before drugs addiction. Similarly, for PDO and mast cell co‐cultures, 100,000 cells of the human mast cell line HMC‐1 or LAD2 will be used.

### 2.9 Flowcytometry

For flow cytometry analysis of B lymphocytes and MC in HGSOC samples, frozen cells are rapidly defrosted in PBS + 2% FBS + 5 mM EDTA (FACS buffer), centrifuged at 1200 rpm for 5 min at room temperature, and stained for 25 min at 4°C in the dark with monoclonal fluorescent conjugated antibodies plus Fixable Viability Dye eFluor 780 (listed in Tables 2, 3, 4), following the manufacturer's instructions. Cells are then washed with 2 mL of FACS Buffer, centrifuged at 1200 rpm for 5 min at room temperature, resuspended in 500uL of FACS Buffer, and acquired with Attune NxT flow cytometer.

**TABLE 2:** List of the antibodies used for flow cytometry analysis of B, T cells, and mast cells, within the TME.

Target	Fluorophore	Clone
CD3	FITC	UCHT1
CD19	eFluor 450	SJ25C1
CD45	PE‐AlexaFluor 610	HI30
CD117 (c‐kit)	APC	104D2
FcεRI	SB600	AER‐37 (CRA1)
Fixable viability dye	eFluor 780	From Invitrogen

**TABLE 3:** List of the antibodies used for flow cytometry analysis of B lymphocytes within the TME.

Target	Fluorophore	Clone
CD19	APC	HIB19
CD20	Brilliant Violet 510	2H7
CD24	PE/Dazzle 594	ML5
CD27	PE/Cy7	O323
IgM	PerCP‐Cy5.5	MHM‐88
CD38	Brilliant Violet 421	HIT2
CD45	FITC	HI30
CD21	SB600	HB5
IgD	SB702	IA6‐2
Fixable viability dye	eFluor 780	From Invitrogen

**TABLE 4:** List of the antibodies used for flow cytometry analysis of circulating B lymphocytes.

Target	Fluorophore	Clone
CD19	APC	HIB19
CD20	Brilliant Violet 510	2H7
CD24	PE/Dazzle 594	ML5
CD27	PE/Cy7	O323
IgM	PerCP‐Cy5.5	MHM‐88
CD38	Brilliant Violet 421	HIT2
CD71	FITC	OKT‐9
IgD	SB702	IA6‐2
Fixable viability dye	eFluor 780	From Invitrogen

Flowcytometry analysis has been performed with FlowJo software.

### 2.10 Histopathology and Immunofluorescence

Biopsy tumor tissue will be fixed in 4% paraformaldehyde, dehydrated, and embedded in paraffin. After antigen removal, sections will be cut. Two biopsy specimens are used for histological examination. and the others will be used for in vitro experiments. The following antibodies will be used: anti‐human CD4, anti‐human CD8, anti‐human CD20 (Dako, Glostrup, Denmark), anti‐human mast cell tryptase (Dako). Aminoethylcarbazole (Dako) is used as a chromogenic substrate. Slides will be evaluated under a Leica DM3000 optical microscope, and microphotographs will be collected with a Leica DFC320 digital camera (Leica, Wetzlar, Germany). All cells will be detected and counted out of 5 × 40 high‐power microscopic fields in each case. For double‐marker immunofluorescence, sections will undergo 2 sequential rounds of single‐marker immunostaining. The following antibodies will be used: anti‐human tryptase, anti‐human TNF‐a, anti‐human IL‐17, anti‐human IL‐10 (all from ThermoFisher) and Alexa Fluor 488– and Alexa Fluor 568–conjugated specific secondary antibodies. Fluorescent images will be collected using a laser scanning confocal microscope (LEICA TCS SP8, Leica Microsystems).

For immunofluorescence analysis of PDO, organoids will be seeded on ibiTreat μ‐Slide 8 wells chambered coverslip (Ibidi) and fixed with 4% PFA (paraformaldehyde) for 20 min at RT. Then wells will be incubated for 20 min with NH_4_Cl at room temperature. Organoids will be permeabilized with PBS + TritonX‐100 0.5% for 10 min at RT, blocked for at least 1 h in PBS + 1% FBS, stained ON with primary antibody and with secondary antibody for 2 h in blocking buffer. The following antibodies will be used: anti‐CK7, anti‐PAX8, anti‐WT‐1 (all from ThermoFisher). Mounting media containing DAPI will be used to stain nuclei.

### 2.11 Statistical Methods

The sample will be described in its characteristics using descriptive statistical techniques. Quantitative variables will be summarized with mean, standard deviation, minimum, and maximum. Qualitative variables will be described with frequency tables (absolute and percentages).

We will record platinum‐free interval, progression‐free survival, overall survival, and sensitivity to platinum‐based chemotherapy.

A univariate and multivariate Cox analysis will be implemented to explore factors associated with progression‐free survival. The proportional hazard assumption will be tested using the Schoenfeld residual test. Multivariable analysis will be corrected for potential confounding factors. Survival curves will be obtained using Kaplan–Meier analysis. All statistical tests will use a two‐tailed significance level of 0.05. The *χ* ^2^ test or Fisher's exact test will be used to analyze the distribution of variables according to different subgroups. The non‐parametric Wilcoxon test will be used to analyze the distribution of continuous variables.

## 3 Discussion

The knowledge of TME, integrated with genetic and molecular features, could help to better understand the OC behavior. In recent decades, considerable effort has been dedicated to understanding the role of immune cells in OC, with particular attention paid to T cells and macrophages. However, therapies targeting T cells (such as those which act on the PD1, LAG3 or TIGIT pathways) have yielded limited success in OC so far [35] suggesting that other immune cells or other pathways might play a pivotal role in this type of tumor.

Among cells under investigation, B cells and mast cells could represent putative crucial cells able to influence the TME. Beyond their antibody‐mediated functions, B cells can act as antigen‐presenting cells influencing T cell activation, can interact with innate immune cells modulating the immune responses in both beneficial and detrimental ways, and can produce mediators with inflammatory or immunosuppressive functions [36].

Similarly, a growing body of literature supports the key role of mast cells, primarily well known for their role in IgE‐mediated allergic response, in tumor progression in a variety of cancers, making them a highly attractive therapeutic target for a wide range of malignancies [37]. However, the data available in the context of gynecological malignancies are mostly controversial, sustaining both pro‐tumoral [38] and anti‐tumoral effect of mast cell on OC [17].

For these reasons, the MICO project aims to identify the main populations of immune cells in the TME and characterize in detail the role of B and mast cells in HGSOC patients by monitoring their accumulation and their phenotype among different patients, in relation to disease progression (FIGO stage) and in response to NACT. In parallel, PDO co‐cultures will be used as an in vitro model to study the effects of B lymphocytes and mast cells on cell growth and drug response. The in vitro data will be compared with patient response to therapy. This information could have both prognostic and therapeutic relevance. Some patients may have tumors with specific TME features that may influence the aggressiveness or treatment response of the tumor itself. From a personalized medicine perspective, the better knowledge of TME could tailor the treatment and management of OC to the unique characteristics of the specific patient's tumor.

Analyses of immunophenotype will assist physicians in identifying those eligible for targeted pharmacological therapies and/or early immunotherapy. Personalized treatment plans will involve combinations of therapies to target both the tumor and its microenvironment.

TME is not static [39]; it can change over time, affecting the tumor's response to treatment. Personalized medicine involves monitoring these changes and adapting the treatment plan as needed. Understanding and targeting the TME is an essential aspect of tailoring cancer treatment to the individual patient's needs and optimizing treatment outcomes. In the future, the results of our study may have implications for the development of effective immunotherapy of epithelial OC, an immunogenic disease currently resistant to checkpoint inhibitors.

A limit of our study might be the heterogeneity of TME in OC and the small size of peritoneal biopsies. To overcome this, biopsies will be collected by an experienced gynecologic oncology surgeon and will be collected where carcinosis will be most represented. If the results in our study are promising, to increase the number of patients and overcome the possible heterogeneity of OC, we will design a multicenter study to validate the results in different populations.

## 4 Conclusions

In this prospective study, we will analyze the association between the composition of the TME, the reactivity of patient‐derived organoids, and patients' clinical data to determine if specific subpopulations of tumor‐infiltrating immune cells can predict epithelial OC outcomes. By comparing molecular profiles, in vitro drug responses, and clinical‐pathological data, we aim to identify patterns that can predict the primary tumor's response and identify patients who may benefit from specific treatments. In the future, our study's results may contribute to developing effective immunotherapy for OC.

## Author Contributions

M.A., E.C., B.F., and G.V. wrote the manuscript. B.F., G.S., S.R., L.M., M.O., L.D., A.T., and C.P. devised the study concept and design. G.V., M.I., M.D.M., and M.A. were responsible for overseeing the statistical section. All authors contributed to the study protocol, read, and approved the final manuscript. Each author has been sufficiently involved in the work to take public responsibility for appropriate portions of the content. Figures and illustrations were designed and created by E.C., S.T., and S.P.

## Disclosure

Patients and the public were not involved in any way in the co‐production process.

## Ethics Statement

This study and all its methods will be carried out in accordance with the ethical standards of the institution and the National Commission for Human Experimentation, as well as the 1964 Declaration of Helsinki and its subsequent amendments or equivalent. This trial has received ethical approval by the Friuli Venezia Giulia Ethics Committee (CEUR FVG) of the Regional Health Institute with ID protocol n. 17 380, in October 2023. This committee is independent and not related to any affiliation of the authors. Any subsequent will of modification of the protocol would be submitted to the agreement of the committee. The clinical trial has been registered Clinical‐Trials.gov with the identifier NCT06272240.

## Consent

The study will be explained to the patients by the gynecologist, and an informed consent form will be obtained from all participants.

## Conflicts of Interest

The authors declare no conflicts of interest.

## Data Availability

The data that support the findings of this study are available from the corresponding author upon reasonable request.
